# Supplementary material for: Deducing subnanometer cluster size and shape distributions of heterogeneous supported catalysts
Source: Nat Commun. 2023 Apr 8;14:1965. doi: 10.1038/s41467-023-37664-w (PMC10082041; doi:10.1038/s41467-023-37664-w)
Supplement: Supplementary file 1 — Supplementary Information [file 41467_2023_37664_MOESM1_ESM.pdf]

Supplementary Information for

Deducing Subnanometer Cluster Size and Shape Distributions of Heterogeneous Supported Catalysts

Vinson Liao[1,2], Maximilian Cohen[1,2], Yifan Wang[1,2], Dionisios G. Vlachos[1,2]

[1]Catalysis Center for Energy Innovation, RAPID Manufacturing Institute, Delaware Energy Institute, 221 Academy St., Newark, DE 19716

[2]Department of Chemical and Biomolecular Engineering, University of Delaware, 150 Academy St., Newark, DE 19716

Table of Contents

Supplementary Note 1: Low-Energy Ensemble via Delaunay Triangulation 2

Supplementary Note 2: Boltzmann Probabilities for Pd_5-Pd_{19} 2

Supplementary Note 3: Effect of Adsorption Site Type and Isomeric Configuration on Primary Spectra 4

Supplementary Note 4: Accounting for DFT Error via Linear Scaling Factors 5

Supplementary Note 5: Error in Bayesian Spectra Deconvolution 6

Supplementary Note 6: Markov Chain Monte Carlo with Stan 7

Supplementary Note 7: Priors for Bayesian Inference 8

Supplementary Note 8: Performance Benchmarking 9

Supplementary Figures 11

Supplementary Tables 19

Supplementary References 20

Supplementary Note 1: Low-Energy Ensemble via Delaunay Triangulation

To enumerate all possible events by finding all available physical adsorption sites, we utilize an algorithm called Delaunay Triangulation (DT) [1]. DT takes a given set of points **P** in space and generates a triangular mesh such that no point in **P** lies inside the circumcircle of any n-dimensional triangle, DT(**P**). We allow the set of points **P** to be the atomic positions of all Pd atoms in a given cluster. All atoms that are located on a triangle whose vertices are not a shared face of multiple tetrahedrons are considered surface atoms. There are many different algorithms with varying run time scalings reported; we choose the one of Lee et al. [2]. Vertices, edges, and centroids of the surface triangles correspond to atop, bridge, and three-fold sites, respectively. Adsorption vectors for atop and bridge sites are computed to maximize the distance away from all atoms, while adsorption vectors for three-fold sites correspond to the normal vector of the plane spanned by the surface triangle pointing away from the cluster. This methodology of enumerating all physical adsorption sites allows for more accurate initial structures for DFT calculations. The structures are ranked from lowest to highest in energy for a given cluster size to determine the low-energy ensemble for direct DFT frequency calculations.

Supplementary Note 2: Boltzmann Probabilities for Pd_5-Pd_{19}

We choose a 95% integrated probability density as a cutoff, as modern FTIR spectrometers with a resolution of 2 cm^{-1} typically have a signal-to-noise ratio (SNR) in the order 400 at the frequencies of the highest observed intensity peaks (i.e., C-O stretch region of 1600-2000 cm^{-1}). The SNR is defined as:

$$SNR = \frac{P_{signal}}{P_{noise}} \tag{S1}$$

where P is the average power measured at equivalent frequencies. Alternatively, we can write the ratio as

$$SNR = \left(\frac{A_{signal}}{A_{noise}}\right)^2 \tag{S2}$$

where A is the root mean square amplitude. For an SNR of 400, the ratio of the amplitudes is 20. We expect 95% of the observed signal to be attributed to the system and 5% to noise. As a result, cluster configurations with Boltzmann probabilities outside the 95% bin contribute intensities indistinguishable from noise. Here, we show the ensemble and Boltzmann probability densities for Pd_5-Pd_{19} at 323 K with saturated CO coverage, as well as the corresponding 95% integrated probability density (**Figure S*1***). The Boltzmann probability for a given configuration, x_i, is computed as follows from statistical mechanics [3]:

$$p(x_i) = \frac{e^{\frac{-\Delta G_i}{k_b T}}}{\sum_{j=1}^{M} e^{\frac{-\Delta G_j}{k_b T}}} \tag{S3}$$

where ΔG_i is the free energy of the adsorbate cluster configuration i, and M and j are the summation over all adsorbate cluster configurations. The ensemble probability and Boltzmann probability density curves are fit using a Gaussian kernel-density and an exponential distribution, respectively. Each point on the curves represents discrete configurations of CO and Pd clusters for a given isomer size. We perform an analogous Boltzmann probability analysis on bare Pd clusters to understand the effect on CO on the number of thermodynamically accessible states (**Figure S*2***). Rather than utilizing the free energy as in Equation (S3) for the calculation, we use the electronic energies from our Hamiltonian, as vibrational contributions to the enthalpy are negligible without adsorbates.

Supplementary Note 3: Effect of Adsorption Site Type and Isomeric Configuration on Primary Spectra

In the main text, we demonstrate the effect of CO coverage on the primary spectra of clusters, and how it is advantageous to operate at saturated coverage regimes for more discernable spectroscopic signatures. At differential coverages, a single high-intensity CO peak exists for each primary spectra; at this coverage, we find that the frequency of the CO peak is highly correlated with the adsorption site-type (i.e., the number of metal atoms the adsorbate is coordinated with) for all cluster sizes Pd_1-Pd_{20}. **Figure S*3*** shows the distributions of CO stretch frequencies versus site type for the aggregated dataset of all cluster sizes. For all cluster sizes, the adsorption site type (i.e., atop, bridge, hollow) almost entirely determines the major spectroscopic peak of our primary spectra. This is mathematically problematic for deconvolution purposes as it leads to an ill-posed problem for traditional frequentist-type statistics, and broad and uninformative posterior distributions for Bayesian-type statistics. This further motivates our decision to operate at the saturated CO coverage regime for the purposes of this work.

In the main text, we have shown examples that clusters of varying sizes at the saturated CO coverage regime. However, we have found that primary spectra of isomers (i.e., of the same cluster size) also exhibit unique spectroscopic signatures and primary spectra, with differences of the same order-of-magnitude as spectra of different cluster sizes. **Figure S*4*** shows an example of the differences between the primary spectra of 4 different isomers of Pd_{10}. Due to the large differences in CO adsorption configurations, it is easy to distinguish between isomers based on their spectroscopic signature.

Supplementary Note 4: Accounting for DFT Error via Linear Scaling Factors

It is customary to apply scaling factors to correct for systematic errors of first-principles computations when compared to experimental results due to errors of the functional used as well as the harmonic approximation of the potential energy surface. In addition, computational modeling of adsorption systems assumes an infinite metal mass approximation. While this approximation reduces the computational cost, it causes calculated frequencies of normal vibration modes driven by adsorbate/metal interactions to be systematically lower than those found experimentally [4]. As a result, we develop linear scaling factors, α, from CO adsorption spectra on Pd single crystals to correct DFT-calculated frequencies.

$$\nu_{EXP} = \alpha \nu_{DFT} \tag{S4}$$

Here, ν_{EXP} is the experimental frequency and ν_{DFT} is the DFT-computed frequency. The experimental frequencies chosen are the frequencies that correspond to the maximum intensity of the experimental spectrum within the C-O stretch frequency range of 1600-2200 cm^{-1}. As the exact overlayer and metal structure are known, we compute their associated C-O stretch frequencies from DFT and calculate a linear scaling factor (and its relative uncertainty) using equations provided by NIST [5]. The details of the DFT calculations are described in the Methods section.

Applying the equations outlined in the methods with the data in **Table S1** results in a scaling factor of 1.017 with a standard deviation of 0.0004. Application of linear scaling factor shifts the DFT computed spectra to better match experimental spectra. We show an example of how scaling factors applied to DFT-computed spectra better match experiments in **Figure S6**.

However, this linear scaling factor only directly applies to adsorption frequencies associated with extended metal surfaces and may not represent the true errors associated with supported single atoms and clusters. We utilize this distribution as a prior distribution as our best estimate for the DFT errors for spectra deconvolution. The "true" linear scaling factor is fit during the Bayesian Inference procedure and utilizes the bounds for the scaling factor on well-defined Pd crystals as a regularization mechanism to prevent overfitting.

Supplementary Note 5: Error in Bayesian Spectra Deconvolution

As described in the Methods section, we model the infrared spectrum, $\vec{y}$, as the vector sum of discretized primary spectra, $\vec{x}_i$, weighted by their relative concentration, c_i, plus some noise, ε:

$$\vec{y} = \sum_{i=1}^{N} c_i \vec{x}_i + \varepsilon,\ \varepsilon \sim \mathrm{N}\ (0, \sigma^2 [\sum_{j=1}^{N} E_j \sum_{i=1}^{N} c_i \vec{x}_i e_j]^2]) \tag{S5}$$

The noise term, ε, is modeled as a multivariate normal distribution with expected value of 0 and covariance $\sigma^2 [\sum_{j=1}^{M} E_j \sum_{i=1}^{N} c_i\, \vec{x}_i e_j]^2$. We claim that σ is equivalent to the reciprocal of the amplitude ratio. Beginning from Equation (S1), we can rewrite the SNR as [6]:

$$SNR = \frac{E[S^2]}{E[N^2]} \tag{S6}$$

where E refers to the expected value, S is the observed signal, and N is random noise. Both the signal and noise must be measured at equivalent points in the system, and in our case, identical frequencies. If the noise has an expected value of 0, i.e., $\mathrm{E}[N] = 0$, we can rewrite the denominator as:

$$E[N^2] = V(N) + [E[N]]^2 = V(N) \tag{S7}$$

where V is the variance. By substituting and rearranging at each frequency, we observe the following:

$$SNR = \frac{E[S^2]}{V(N)} = \frac{S^2}{V(N)} = \frac{(\sum_{i=1}^{N} c_i \vec{x}_i)^2}{\sigma^2 [\sum_{j=1}^{N} E_j \sum_{i=1}^{N} c_i \vec{x}_i e_j]^2_i} = \frac{1}{\sigma^2} = \left(\frac{A_{signal}}{A_{noise}}\right)^2 \tag{S8}$$

The error parameter, σ, is equivalent to the reciprocal of the amplitude ratio.

Supplementary Note 6: Markov Chain Monte Carlo with Stan

We utilize the open-source Bayesian inference software PyStan, a Python interface to Stan, to estimate the posterior distribution of our model parameters. Markov Chain Monte Carlo (MCMC) is the most robust sampling algorithm offered by Stan for approximating the posterior [7]. MCMC estimation obtains samples of the model parameters that generates a histogram that accurately presents the posterior. The sampling methodology occurs through a series of Markov transitions that collect samples distributed indistinguishably from the posterior. The Markov chain generated will converge towards the true posterior as the number of samples collected tends towards infinity, assuming the sampling condition is met, regardless of the initial point of the sampling. Stan ensures the sampling condition is met using a Hamiltonian Monte Carlo (HMC) approach.

The HMC approach transforms the N-dimensional parameter space into an (N+1)-dimensional potential energy surface, where each coordinate of the surface is defined by a unique set of parameter values. This transformation does not require the calculation of the marginal. Sets of parameters with high probabilities map to low potential energies and vice versa. A starting point

in this potential energy surface and a constant total system energy are chosen, and a fictitious object navigates this potential energy landscape to generate optimal samples along the object trajectory. Coordinates with low potential energy and high probabilities are more likely to be traversed by this fictitious object. The No-U-Turn Sampler (NUTS) is applied to determine optimal trajectories. NUTS extends trajectories in both the forward and reverse directions along the potential energy surface until a U-turn is made. This prevents the samples from being generated in a space that has already been explored. The HMC approach, combined with NUTS, is a highly efficient method of sampling the posterior. With enough samples, this output can accurately approximate the posterior distribution of our model parameters and allows for accurate computations of relevant statistical quantities of interest, such as the distribution mean and standard deviation.

Supplementary Note 7: Priors for Bayesian Inference

Choosing appropriate prior distributions for our model parameters is important as it affects the calculated posterior distribution [8]. There are three major classifications of priors used in the literature: informative, weakly informative, and diffuse. These classifications are usually based on the researchers' expert knowledge and prior experience with the system. For spectral deconvolution, we have 3+N unique model parameters, 3 of which are related to properties of the primary spectra, and N cluster fraction parameters. In order to construct primary spectra, 3 parameters are needed that affect the final spectral shape and intensities: full-width half-maximum (FWHM), fraction Lorentzian (fL), and linear scaling factor (α). The shape parameters, FWHM and fL, are associated with spectral broadening that occurs in experimental spectra. Spectral broadening is mainly associated with lifetime broadening, thermal broadening, and pressure broadening [9]. No physical models currently exist to model these phenomena for

metal adsorbates, so we choose a diffusive prior to describe them. There have been efforts to study intrinsic peak widths of adsorbed CO through single crystals with well-defined overlayers as the obtained spectra have minimal overlapping peaks due to uniformity in adsorbates, but the applicability to clusters remains unknown [10]. The linear scaling factor, α, corrects for errors in computed frequencies due to the harmonic approximation and utilizes a weakly informative prior derived in previous sections of the Supplementary Information using data collected from UHV experiments. Finally, for the N cluster fraction parameters, we use a diffuse prior, as no prior information is known about the system at hand. Information about cluster size fractions from other spectroscopy or microscopy-based characterization used in tandem can be leveraged to hypothesize a weakly informative or informative prior.

Supplementary Note 8: Performance Benchmarking

In the main text, we demonstrated the ability of our Bayesian deconvolution method in predicting subnanometer cluster size distributions directly from both synthetic and experimental spectra. Here, we show the efficacy of our methodology on 100 synthetic spectra with varying artificial Gaussian noise constructed following the Methods section and Equation S5. Both the relative concentrations of each species from Pd_1-Pd_{20} as well as the extent of Gaussian noise for each spectrum are randomized (these values are noted as ground truths for benchmarking purposes). Noise is simulated with signal-to-noise ratios ranging from infinity (e.g., infinitesimally small noise, the limit as σ approaches 0) to 25 (e.g., the lowest SNR of FTIR receivers reported in the literature, the limit as σ approaches 0.2) by uniformly sampling values of $0<\sigma<0.20$. Note both bounds of signal-to-noise ratios are unrealistic to expect for observed experimental spectra obtained from modern-day FTIR receivers and purely serve as limits for benchmarking. We show a parity plot of the predicted cluster size fractions, binned in three

categories (single-atoms for Pd_1, 2D clusters for Pd_2-Pd_5, and 3D clusters for Pd_6-Pd_{20}) as in the main text, as a function of the true value of σ for each spectrum in **Figure S*5***. As the Bayesian deconvolution of each spectrum yields a distribution of sampled values, we utilize the maximum a posteriori (MAP) estimation, or the mode of the distribution, as point estimates for the parity plot. The MAP estimate and mean of the distributions were found to be essentially identical over 100 spectra. For 100 spectra, the deconvolution procedure yields a mean absolute error of 0.049 for the predicted cluster fractions over 300 data points (as each spectrum yields 3 predictions) and the entirety of the range of simulated noise values. Surprisingly, the prediction error is not correlated with the amount of the simulated noise for the SNR values studied, corresponding to $0<\sigma<0.20$. This is a good indication that the model is robust enough to avoid overfitting spectra to noise. More importantly, however, is that for all 100 spectra, the true cluster fraction lies within the 95% credible interval of the distribution.

Supplementary Figures

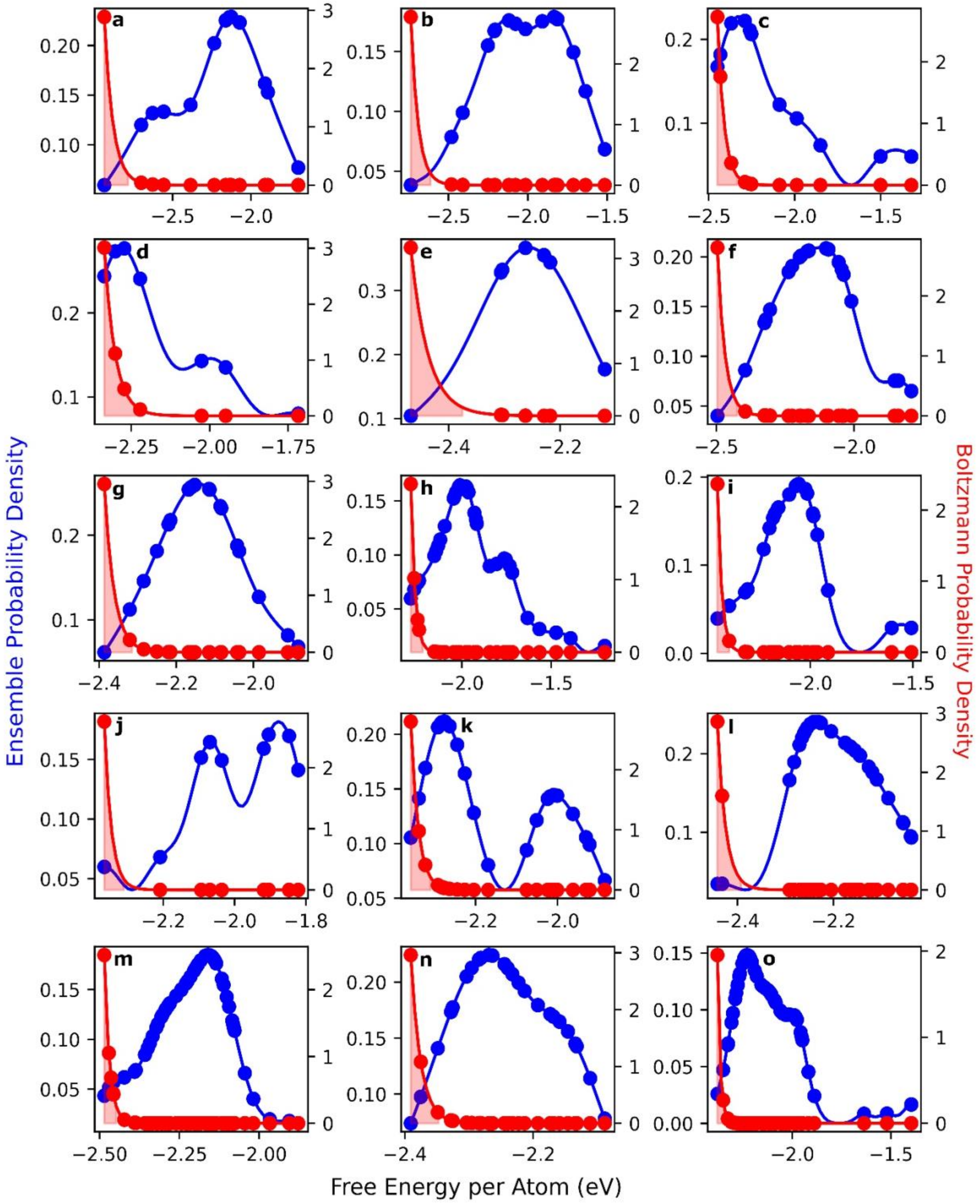

Figure S1. **Ensemble and Boltzmann probabilities for (a) Pd_5 - (o) Pd_{19}/CeO_2 at 323K saturated with CO.** Each point along the probability density curves represents a discrete

$(CO)_m/Pd_n$ configuration. The ensemble probability density assumes each discrete state is equally probable, whereas the Boltzmann probability density weights each discrete state by its respective Boltzmann factor. The shaded red region represents the integrated 95% probability density. At low temperatures, relatively few discrete states are energetically accessible and dominate the ensemble of isomers compared to high temperatures. The number of discrete states that meet the 95% cutoff criteria range from 1 to 4 states over the range of Pd_5-Pd_{20}.

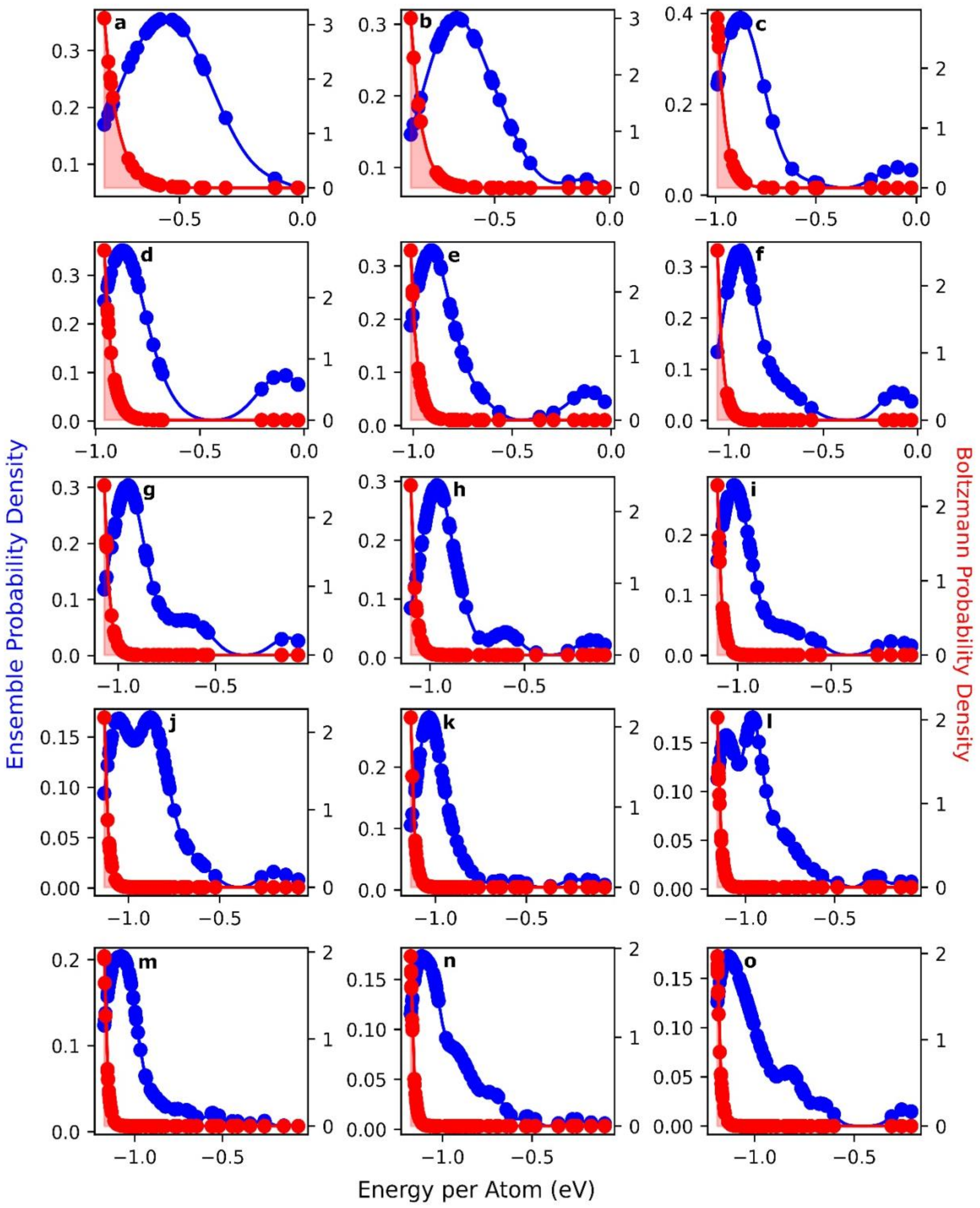

Figure S2. Ensemble and Boltzmann probabilities for bare (a) Pd_5 - (o) Pd_{19}/CeO_2 at 323K.

Each point along the probability density curves represents a discrete Pd_n configuration. The

ensemble probability density assumes each discrete state is equally probable, whereas the Boltzmann probability density weights each discrete state by its respective Boltzmann factor. The shaded red region represents the integrated 95% probability density. At low temperatures, relatively few discrete states are energetically accessible and dominate the ensemble of isomers compared to high temperatures. The number of discrete states that meet the 95% cutoff criteria range from 10 to 40 states over the range of Pd_5-Pd_{20}.

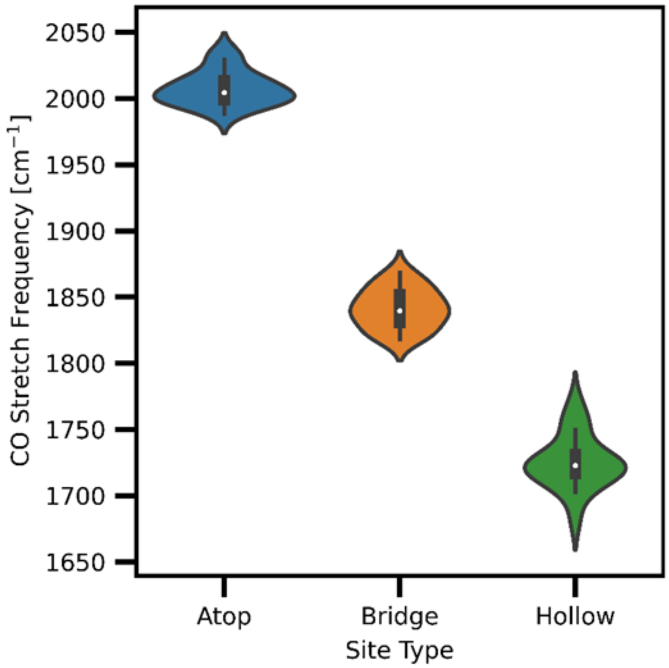

Figure S3. CO stretch frequencies at differential coverage versus site type. Each violinplot shows the distribution of frequencies of the aggregated data for all cluster sizes of Pd_1-Pd_{20}. The width of the curves corresponds to the relative probability density of observing a given CO stretch frequency. We find that at differential coverage, the CO stretch frequency is highly correlated with the site type, which makes the primary spectra generated mathematically difficult to utilize for deconvolution purposes.

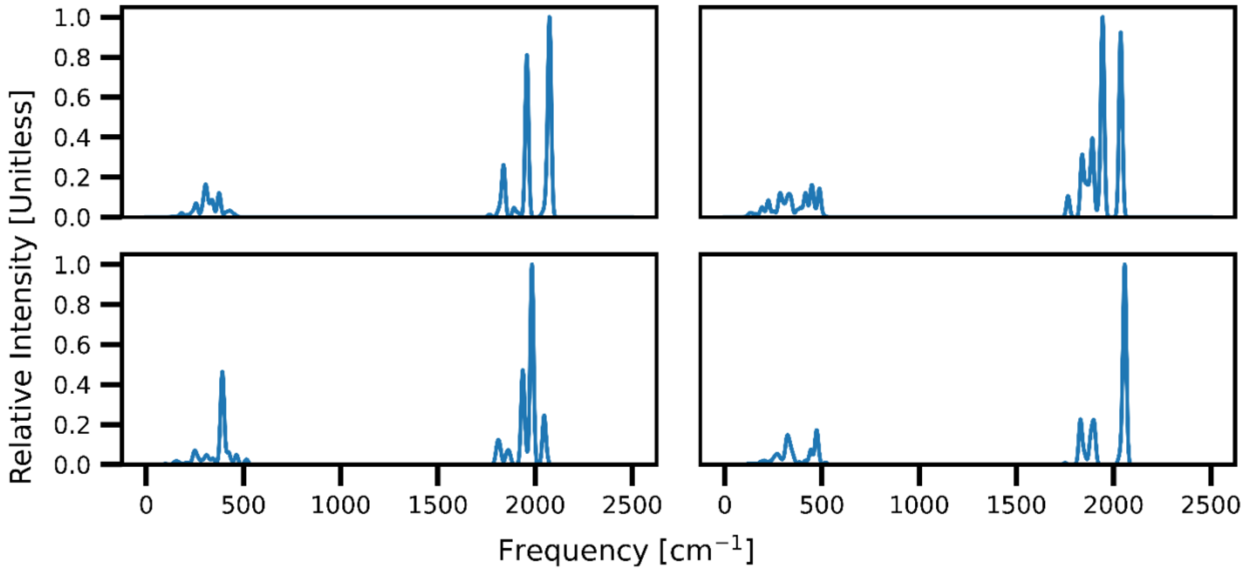

Figure S4. Primary spectra of saturated CO on various isomers of Pd_{10}/CeO_2 from DFT-computed frequencies and intensities. The spectroscopic signature of different isomers of the same cluster size exhibit largely different spectroscopic signatures, similar to the differences between primary spectra of different cluster sizes, due to highly different CO adsorbate configurations. The intensities of the metal-carbon stretch region (< 1000 cm^{-1}) are magnified tenfold for visibility.

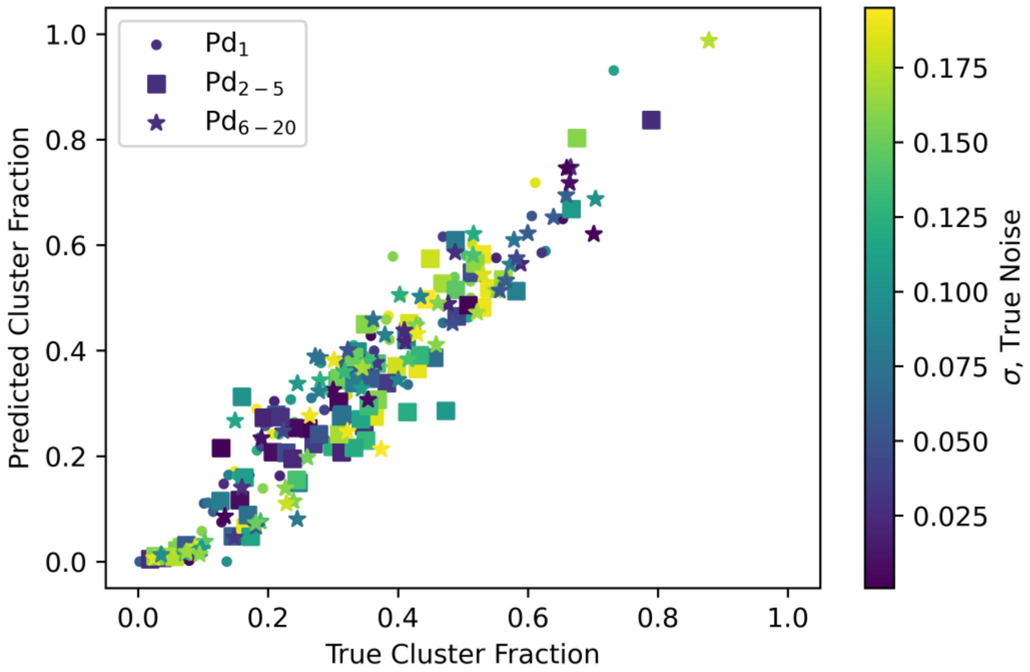

Figure S5. Parity plot of predicted cluster fractions from Bayesian deconvolution of 100 synthetic spectra with varying simulated noise. Cluster fractions and simulated noise is randomized from a uniform distribution of 0<ci<1 (followed by multiplication by a normalization constant) and $0<\sigma<0.20$, respectively. MAP estimates of posterior distributions, which are identical to the means, are used as point estimates. The mean absolute error (MAE) in the predicted cluster fractions is 0.049. The predicted error is uncorrelated to the amount of simulated noise in the system for the values studied.

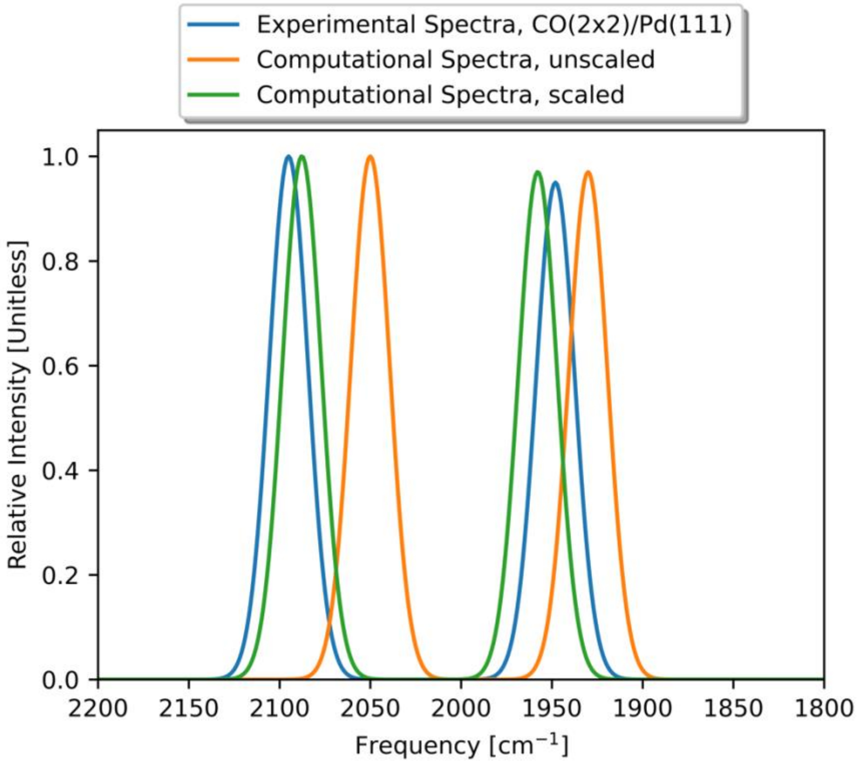

Figure S6. Comparison of experimental IRAS[11] versus DFT-computed spectra for CO on Pd(111) in a (2x2) configuration. Plotted is the relative intensity versus the wavenumber. DFT-calculated frequencies were convoluted with a Gaussian function with full width half maximum of 25 cm^{-1}. The scaled spectra show much better agreement with the experimental spectra, especially when comparing the atop bound peak in the 2100 cm^{-1} range. Linear frequency scaling factors are effective in minimizing systematic errors associated with DFT-computed frequencies.

Supplementary Tables

Pd Facet	Overlayer Structure	Absolute Coverage [ML]	Binding-Type	Experimental [cm^{-1}]	DFT [cm^{-1}]
(111)	Singleton	0.07	hollow	1813[12]	1692
(111)	(2x2)	0.75	atop	2095[12]	2050
(111)	(2x2)	0.75	bridge	1948[12]	1930
(111)	c(4x2)	0.50	bridge	1948[12,13]	1912
(111)	(√3x√3)R30°	0.33	bridge	1836[14]	1857
(100)	c(2√2x√2)R45°	1.00	bridge	1964[15]	1935
(100)	c(2√2x√2)R45°	0.05	bridge	1895[15]	1890

Table S1. Experimental and DFT C-O stretch frequencies from spectra with well-characterized coverages and overlayers. We utilize these values to generate linear scaling factors to correct for errors in DFT-calculated frequencies. We calculate a scaling factor of 1.017 with an uncertainty of 0.0004.

Supplementary References

1. Cazals, F. & Giesen, J. Delaunay Triangulation Based Surface Reconstruction. in *Effective Computational Geometry for Curves and Surfaces* (eds. Boissonnat, J.-D. & Teillaud, M.) 231–276 (Springer Berlin Heidelberg, 2006). doi:10.1007/978-3-540-33259-6_6.
2. Lee, D. T. & Schachter, B. J. Two algorithms for constructing a Delaunay triangulation. *International Journal of Computer and Information Sciences* **9**, 219–242 (1980).
3. McQuarrie, D. A. *Statistical mechanics*. (New York, 1976).
4. Black, J. E., Rahman, T. S. & Mills, D. L. Electron energy loss spectroscopy of adsorbed atoms. *Journal of Vacuum Science and Technology* **20**, 567–573 (1982).
5. CCCBDB Vibrational frequency scaling factors. https://cccbdb.nist.gov/vibnotes.asp.
6. Butler, J. L. & Sherman, C. H. *Transducers and Arrays for Underwater Sound*. (Springer International Publishing, 2016). doi:10.1007/978-3-319-39044-4.
7. Chemical Kinetics Bayesian Inference Toolbox (CKBIT) - ScienceDirect. https://www.sciencedirect.com/science/article/abs/pii/S0010465521001016.
8. Sarma, A. & Kay, M. Prior Setting in Practice: Strategies and Rationales Used in Choosing Prior Distributions for Bayesian Analysis. in *Proceedings of the 2020 CHI Conference on Human Factors in Computing Systems* 1–12 (Association for Computing Machinery, 2020).
9. Myers, A. B. Molecular electronic spectral broadening in liquids and glasses. *Annual Review of Physical Chemistry* **49**, 267 (1998).
10. Kenjiro Yoshioka, Fusao Kitamura, Mitsuaki Takeda, Machiko Takahashi, & Masatoki Ito. Infrared reflection absorption spectra of carbon monoxide adsorbed on single crystal electrodes, Pd(111) and Pd(100). *Surface Science* **227**, 90–96 (1990).

11. Szanyi, J., Kuhn, W. K. & Goodman, D. W. CO adsorption on Pd(111) and Pd(100): Low and high pressure correlations. *Journal of Vacuum Science & Technology A: Vacuum, Surfaces, and Films* **11**, 1969–1974 (1993).
12. Hoffmann, F. M. Infrared reflection-absorption spectroscopy of adsorbed molecules. *Surface Science Reports* **3**, 107–192 (1983).
13. Conrad, H., Ertl, G., Koch, J. & Latta, E. E. Adsorption of CO on Pd single crystal surfaces. *Surface Science* **43**, 462–480 (1974).
14. Bradshaw, A. M. & Hoffmann, F. M. The chemisorption of carbon monoxide on palladium single crystal surfaces: IR spectroscopic evidence for localised site adsorption. *Surface Science* **72**, 513–535 (1978).
15. Ortega, A., Huffman, F. M. & Bradshaw, A. M. The adsorption of CO on Pd(100) studied by IR reflection absorption spectroscopy. *Surface Science* **119**, 79–94 (1982).
